# Prevalence and associated factors of depression and anxiety among Sudanese refugees at Bambasi Camp in northwest Ethiopia: a cross-sectional study

**DOI:** 10.3389/fpsyt.2024.1505876

**Published:** 2024-12-19

**Authors:** Endris Seid Amede, Elias Tesfaye, Gutema Ahmed

**Affiliations:** ^1^ Department of Psychiatry, College of Health Science and Medicine, Dilla University, Dilla, Ethiopia; ^2^ Psychiatry Department, Faculty of Medicine, Institute of Health, Jimma University, Jimma, Ethiopia

**Keywords:** depression, anxiety, Sudanese refugee, Ethiopia, Bambasi Camp

## Abstract

**Background:**

Refugees encounter a variety of traumatic events throughout their migratory process and in the camp, which increase their risk of developing mental illnesses. Even though depression and anxiety are the most frequent after a stressful life event, there is limited information on Sudanese refugees. Therefore, this study aimed to assess the prevalence and associated factors of depressive and anxiety symptoms at the Bambasi Camp in northwestern Ethiopia.

**Method:**

A cross-sectional study was conducted among 379 participants using a systematic random sampling method. The Hopkins Symptoms Checklist (HSCL-25) was used to assess depressive and anxiety symptoms. The data was collected by the Kobo toolbox mobile application and analyzed using SPSS (version 26). Multiple logistic regressions with the backward elimination method were performed. A *p*-value of <0.05 with 95% CI was taken as statistically significant.

**Result:**

A total of 379 participants were included, with 96.9% response rate. The prevalence of depressive and anxiety symptoms was 46.2% (95% CI = 41–51.2) and 39.6% (95% CI = 34–41), respectively. A multivariable logistic regression analysis indicated that being a female (AOR = 2.56; 95% CI = 1.50–4.26), duration of stay (≥11 years) in the camp (AOR = 2.32; 95% CI = 1.39–3.86), being jobless (AOR = 2.68; 95% CI = 1.30–5.50), and poor social support (AOR = 3.12; 95% CI = 1.25–7.79) were identified as risk factors of depressive symptoms and also being female (AOR = 3.60; 95% CI = 2.26–5.74) and age above 45 (AOR = 2.48; 95% CI = 1.16–5.30) were identified as risk factors for anxiety symptoms.

**Conclusion:**

The findings highlight the high burden of mental health problems that Sudanese refugees bear. The identified predictors of depressive and anxiety symptoms should alert medical and refugee professionals to identify vulnerable individuals and groups, to link them to appropriate psychological intervention, and also to take action for the identified risk factors.

## Introduction

According to the World Health Organization (WHO), depression is the leading cause of disability, with an estimated 264 million people affected globally (1). It is the third most common cause of years lived with disability (YLDs) in the general population (2). Anxiety is an ongoing feeling of worry or apprehension that is inappropriate given the circumstances of one’s life. It can be expressed in different ways, such as uncontrollable worry, intense fear, phobias, and panic attacks (3). In 2017, 260 million people globally were affected by anxiety disorder (4).

The latest figures show that the number of conflict-affected people reached more than 100 million, which is an increase due to recent global events (5). According to the United Nations High Commissioner for Refugees (UNHCR) reports, by the end of 2021, 27.1 million refugees were in refugee camps, 83% were hosted in low- and middle-income countries, and one-fifth of all refugees worldwide are hosted in Africa, predominantly in three countries: Uganda, Sudan, and Ethiopia (6). During the conflict between Sudan and South Sudan in 2013, nearly 400,000 people died, 4 million people were displaced (2 million within Sudan) and another 2.5 million were refugees in neighboring countries like Ethiopia (7).

Nearly all individuals affected by these emergencies will experience psychological distress. An estimated 35 million (42%) of the 82.4 million displaced and stateless people globally experience mental disorders such as depression and anxiety (8). Refugees and internally displaced people had a reported prevalence of post-traumatic stress disorder, depression, and nonspecific anxiety disorders that varied widely from 3% to 88%, 5% to 80%, and 20.3% to 81%, respectively (9). A meta-analysis of studies on long-term mental health with a total of 16,010 war-affected refugees showed that the prevalence of depression was at 2.3–80%, of PTSD at 4.4–86%, and of anxiety disorder at 20.3–88% (10). Recent research found that among 43% of Syrian refugees living in 10 countries, 40% had depression and 26% had anxiety (11). In Africa, the prevalence of depression was 49.9%, 32%, 48.1%, 37.8%, and 45% in southern Sudan, Rwandan refugees in Uganda, and Somali refugees, in Maiayni camp and Dabat town, Ethiopia, respectively (12–15), while the prevalence of anxiety was 49.4% in South Africa (16), 73% in Uganda (17), and 33.6% in Ethiopia (15).

The greater susceptibility of migrants to depression has been connected to both pre-migration events, like exposure to war trauma (18), and post-migration adverse circumstances that refugees frequently encounter in a new country, such as persecution, violence and human right violations (19) and also being separated from family, having trouble with the asylum procedure or even being detained, being unemployed, and having problems assimilating, losing relatives, sexual and physical assault, and lack of food or shelter (20). Moreover, they are underprivileged and vulnerable groups, whose vulnerability is due to the fact that they lack the same legal protections as citizens, making them vulnerable to abuse, hostility from the community, and economic disadvantage, all of which are risk factors for psychological distress (21). Delayed access to mental health care after resettlement increases the risk of depression more than fourfold in traumatized refugees exposed to adverse life experiences compared to the general population (22).

Even though the majority of refugees live in low-income countries, research on refugee mental health is overwhelmingly limited to those who reside in western countries (23). Currently, there is a scarcity of information on the prevalence of depression, anxiety, and their associated factors in the Bambasi refugee’s camp. To address this critical information gap, our study aims to assess the prevalence and its associated factors of depression and anxiety among Sudanese refugees residing in Bambasi Camp, northwest Ethiopia.

This study contributes to the field of existing evidence on mental health issues among refugees, particularly in Ethiopia, and gives insight into the mental health needs of this vulnerable population. It can also inform policymakers and aid organizations about the development of policies and programs that address the mental health needs of refugees and provide the necessary support. Moreover, the study has practical implications for mental health professionals, counselors, social workers, and aid workers working with refugees by identifying those associated factors and providing targeted interventions that address the specific mental health needs.

## Materials and methods

### Study area and period

A refugee-camp-based cross-sectional study was conducted from August 1 to 30, 2023 in Bambasi Refugee Camp, northwest Ethiopia. It is found in Benishangul Gumuz Regional State, 661 km away from Addis Ababa, which is the capital city of Ethiopia. The camp is located in Womba Kebele, 48.6 km from Assosa town, which is the capital city of the region. The camp was established in 2012 as a temporary home for Sudanese refugees. As of November 2020, UNHCR reports that the camp has 4,085 registered households and hosts 18,296 refugees, of whom 9,149 are female (24). The camp is divided into three zones, namely, zone A, zone B, and zone C. It has one health center jointly funded by the United Nations High Commissioner for Refugees (UNHCR) and the Agency for Refugees and Returnee Affairs (ARRA). It provides several health services, including mental health services. Currently, they mainly depend on monthly aid.

### Population

All adults who settled in the Bambasi refugee camp were source of population.

#### Study population

All randomly selected individuals age 18 years and above were from selected households.

### Eligibility criteria

All adults living in the Bambasi refugee camp during the study period were included in this study. Participants who were unable to provide information due to illness during the data collection period were excluded from the study

### Sample size determination

A single population proportion formula was used to calculate the sample size. The sample size was determined by taking the assumption that, based on a research conducted among Eritrean refugees in the Tigray region, the proportion of depression was 37.8% (14) and by considering 95% confidence level (*zα*/2 = 1.96) and 5% marginal error (*d* = 0.05). Therefore, the sample size was calculated by using the following formula:


$$n=\frac{{\left(Z_{\alpha /2}\right)}^{2}\times \;pq}{d^{2}}\;\mathrm{where}\;n=\;\mathrm{sample}\;\mathrm{size}$$

*q* = 1 - *p*. Thus, (1.96)^2^ (0.388) (1 - 0.388)/(0.05)^2^ **=** 355, and by considering 10% non-response rate, the final sample size was = 391.

### Sampling technique

A systematic random sampling method was employed to select 379 individual households from a total of 4,085 households. The camp has three zones; proportional allocation of the sample size to each zone was done. The interval (K) was calculated by dividing the number of households by the sample size allocated to the specific zone and was found to be 10. Then, every k (10) value was used to select a HH from each zone. The first household was selected by a lottery method, and then the next household was selected by adding 10 to the first household. The lottery method was used to select study participants within the household if there were more than one.

### Data collection procedure

The questionnaire was first prepared in English, then translated to Arabic (the local language) by fluent language speakers, and again translated back to English by another person to ensure consistency and understandability. Next, three BSc nurses for data collection and one MSc in psychiatry were recruited for supervision. The data collectors and supervisor received training on the data collection tools, procedures, and ethical concerns. The questionnaire was pre-tested before the actual study was conducted. Close supervision was carried out during the data collection period, and the investigators checked the consistency of the completed questionnaire at the end of each day. After that, data collection was performed through a face-to-face interview using a pre-tested structured and standardized questionnaires by Kobo Toolbox mobile application.

### Instruments

#### Depression and anxiety symptoms

The Hopkin Symptom Checklist (HSCL-25) is a symptom inventory that is used to assess symptoms of anxiety and depression. It has 25 items: Part 1 consists of 10 items for anxiety and Part 2 has 15 items for depression. Each question has four response categories on the following scale: “not at all,” “a little,” “quite a bit,” and “extremely,” which are rated 1 to 4, respectively (25). The depression score is the average of 15 depression items, while the anxiety score is the average of 10 anxiety items. The overall score has a strong correlation with severe emotional distress of an unspecified anxiety diagnosis, as demonstrated by numerous refugee groups, and the depression score is comparable with major depression as described by the Diagnostic and Statistical Manual of the American Psychiatric Association, fourth edition (DSM-IV). This study was conducted with the Arabic version of the scale, which had been used and proven to be valid and reliable by previous studies (26). It is also validated in Africa (27) and used in multiple internal displaced and refugee populations in sub-Saharan countries including Ethiopia, Kenya, Uganda, and Sudan (28, 29). For the current study, the Cronbach alpha value was 0.81.

#### Social support

Oslo Social Support (OSS-3) is a brief assessment of social support and functioning. It consists of three items that ask for the number of close confidants, the attention and concern shown by others, and the ease of getting practical aid from others, covering different aspects of social support. The total scores are calculated by summing up the raw scores for each item. The sum of the raw scores has a range of 3–14. The scores “3–8” indicate poor social support, “9–11” indicate moderate social support, and “12–14” indicate strong social support (30).

#### Substance use history

Risky substance use-related factors are assessed by using the Alcohol, Smoking, and Substance Involvement Screening Test (ASSIST), which is a brief screening questionnaire developed and validated by the World Health Organization (WHO) (31).

### Variables of the study

Dependent variables: depressive and anxiety symptoms (yes/no)

Independent variables: socio-demographic characteristics such as sex, age, marital status, educational status, length of stay in the camp, and occupational status

Behavioral- and social-related factors included risky alcohol, tobacco, khat, other substance use, and social support status.

Clinical-related factors included past psychiatric history, family history of mental illness, and chronic medical illness.

### Operational definition

Depressive symptoms: If the participants are scoring a mean score of ≥1.75 out of the potential 4 points on the depression subscale of HSCL-25, they are categorized as experiencing depressive symptoms, and those who scoring <1.75 have no depressive symptoms (26). 

Anxiety symptoms: If the participants have a mean score of ≥1.75 on the anxiety subscale of HSCL-25, they have anxiety, and for those with a score <1.75, they have no anxiety symptoms (26).

Level of social support: Participants who scored 3–8 had poor social support, 9–11 had moderate social support, and 12–14 had strong social support (30).

Risky alcohol use: Participants scoring 0–10, 11–26, and ≥27 from the ASSIST questionnaire have low, moderate, and high risk, respectively (31).

Risky substance use (khat, cigarettes, and other substances): Those participants scoring 0–3, 4–26, and ≥27 from the ASSIST questionnaire had low, moderate, and high risk, respectively (31).

### Data quality control

A 1-day training was given to the data collectors and supervisor on the objectives, clarity of tools, and overall data collection procedures to standardize the interview procedures and reduce the interviewer bias. The final version of the questionnaire was translated into Arabic and again retranslated into English language by bilingual experts to check for the language consistency of the tool. The questionnaires were pre-tested on 5% (20 refugees) of the sample size to determine the clarity of the tool and the feasibility of the study. At the end of each day during the data collection period, the collected data were checked for consistency and completeness.

### Data processing and analysis

The collected data were checked for completeness and consistency, edited and coded by using the Kobo toolbox application, and then exported to SPSS version 26 statistical software for analysis. Descriptive statistics such as frequency and percentage were used to summarize the variables and were presented using charts and tables. Bivariate logistic regression analysis was done separately with outcome variables, and all explanatory variables that have an association with the outcome variables with *p <*0.25 were selected as candidate variables for multivariable logistic regression analysis. A multivariable logistic regression analysis with backward elimination was carried out. Finally, variables with *p <*0.05 and 95% confidence interval and odds ratio (OR) are considered as factors associated with the outcome variables (statistically significant). The *p*-values in the Hosmer and Lemshow tests were 0.69 and 0.64 for anxiety and depression, respectively.

## Results

### Socio-demographic characteristics of the study participants

Out of 391 selected households in the study, 379 individuals participated, yielding a response rate of 96.9%. Of the 379 participants, more than half (207, 54.6%) were female. Their age ranged from 18 to 74 years; 127 (33.5%) were between 26 and 35 years of age. More than one-third (149, 39.3%), and almost all (367, 96.8%) were married and Muslim by religion, respectively. More than one-third of the participants (143, 37.7%) had no formal education. The average duration of stay in the camp was 11.4 years, ranging from 9 to 12 years. Although the participants had diverse types of occupations before coming to Ethiopia, farmers constituted one-third (32.2%) of the total number of participants (**Table 1**).

**Table 1:** Socio-demographic characteristics of participants among Sudanese refugees at Bambasi Camp in northwest Ethiopia, 2023 (*n* = 379).

Variable	Category	Frequency	Percentage
Sex	Male	172	45.4
	Female	207	54.6
Age	18–25	102	26.9
	26–35	127	33.5
	36–45	104	27.4
	>45	46	12.2
Marital status	Married	149	39.3
	Single	114	30.1
	Separated	69	18.2
	Divorced	23	6.1
	Widowed	24	6.3
Educational status	No formal education	143	37.7
	Primary	129	34
	Secondary school	79	20.8
	College and above	28	7.4
Occupation in Sudan	Government employee	100	26.4
	Farmer	122	32.2
	Housewife	53	14
	Student	29	7.7
	Self-employed	75	19.8
Duration of stay in camp	<11 years	181	47.8
	≥11 years	198	52.2
Occupation in Ethiopia	Yes	62	16.4
	No	317	83.6

### Clinical-related factors

Among the participants, 14.2% had chronic medical condition (**Table 2**).

**Table 2:** Frequency distribution of clinical-related factors among Sudanese refugees at Bambasi Camp in northwest Ethiopia, 2023 (*n* = 379).

Variables	Category	Frequency	Percentage
PTSD symptoms	No	219	57.8
	Yes	160	42.2
Chronic medical illness	No	325	86.8
	Yes	54	14.2
Past mental illness	No	357	94.2
	Yes	22	5.8
Family mental illness	No	327	86.3
	Yes	52	13.7

### Substance- and psychosocial-related factors

More than half of the participants [206 (54.4%), 168 (44.3%), and 140 (36.9%)] had used khat, alcohol, and cigarette in their lifetime, respectively. Additionally, one out of seven (54, 14.2%) participants had had risky alcohol use behavior. Regarding social support status, nearly half (177, 46.7%) of the participants had poor social support (**Table 3**).

**Table 3:** Frequency distribution of risky substance use and psychosocial-related factors among Sudanese refugees at Bambasi Camp in northwest Ethiopia, 2023 (*n* = 379).

Variables	Category	Frequency	Percentage
Risky khat use	Low	232	61.2
	Moderate	117	30.9
	High risk	30	7.9
Risky alcohol use	Low risk	288	76
	Moderate	37	9.8
	High risk	54	14.2
Risky tobacco use	Low risk	271	71.5
	Moderate	59	15.6
	High risk	49	12.9
Other substances^a^	Low risk	364	96
	Moderate	6	1.6
	High risk	9	2.4
Social support	Good	37	9.8
	Moderate	165	43.5
	Poor	177	46.7

^a^Cannabis and inhalant.

### Prevalence of depressive and anxiety symptoms

Among the participants, 175 (46.2%, 95% CI = 41–51%) had depression, with more women than men [116 (66.3%) vs. 59 (33.7%)], fulfilling the mean score of ≥1.75 from HSCL-15, and

**Figure 1:**
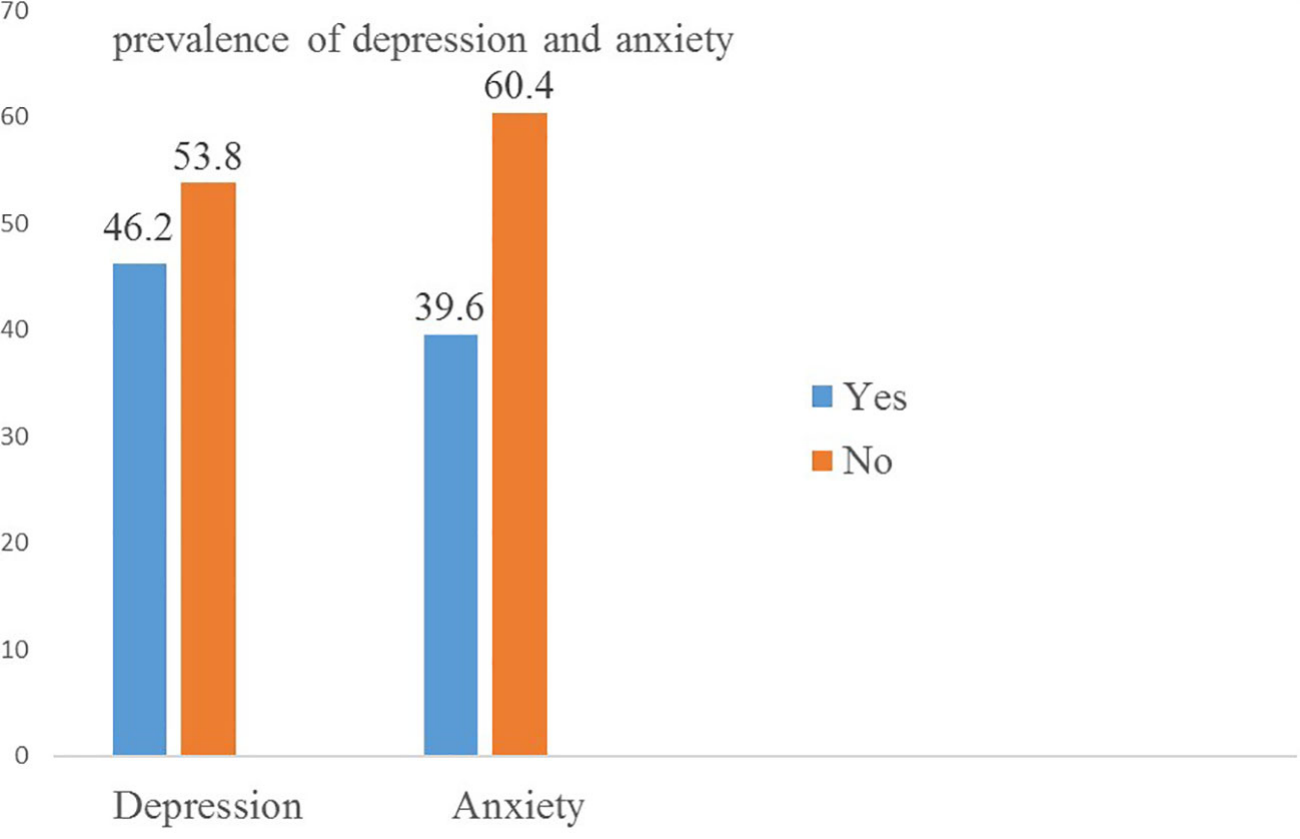
Prevalence of depression and anxiety among Sudanese refugees at Bambasi Camp in northwest Ethiopia, 2023 (*n* = 379).

150 (39.6%, 95% CI = 35–45%) had anxiety, fulfilling the mean score of ≥1.75 from the HSCL-10 anxiety subscale (**Figure 1**).

### Factors associated with depressive symptoms

Among the factors, age, sex, educational status, marital status, job status in Ethiopia, family history of psychiatric illness, presence of chronic medical illness, and social support fulfilled the minimum criteria (*p* < 0.25) for further multivariable logistic analysis. These variables were then entered into a multivariable logistic regression model (**Table 4**).

**Table 4:** Bivariate and multivariable analysis for all candidate factors for depression among Sudanese refugees at Bambasi Camp in northwest Ethiopia (*n* = 379).

Variable	Category	Depression		COR and 95% CI	p-value	AOR and 95% CI	p-value
		Yes	No				
Sex	Male	59 (34.3)	113 (65.7)	1		1	
	Female	116 (56%)	91 (44%)	2.44 (1.60, 3.70)	.001	2.55(1.50, 4.26)	.000
Job in Ethiopia	Yes	15 (24.2%)	47 (75.8%)	1		1	
	No	160 (50.5%)	157 (49.5%)	3.19 (1.72, 5.95)	.000	2.68 (1.30, 5.50)	.007
Duration of stay in camp	≤10 years	67 (37%)	114 (63%)	1			
	≥11 years	108 (54.5%)	90 (45.5%)	2.04 (1.35, 3.08)	.001	2.32 (1.39, 3.87)	.001
Social support	Strong	9 (24.3%)	28 (75.7%)	1		1	
	Moderate	61 (37%)	104 (63%)	1.83 (0.81, 4.12)	.148	1.57 (0.62, 3.96)	.339
	Poor	105 (59.3%)	72 (40.7%)	4.54 (2.02, 10.18)	.000	3.12 (1.25, 7.79)	.015
Chronic illness	No	146 (45%)	179 (55%)	1		1	
	Yes	29 (53.7%)	25 (46.3%)	1.42 (0.79, 2.54)	.232	1.44 (0.67, 3.09)	.354
Past mental illness	No	162 (45.4%)	195 (54.6%)	1		1	
	Yes	13 (59.1%)	9 (41.9%)	1.74 (0.73, 4.17)	.215	1.65 (0.54, 5.02)	.375
Family mental illness	No	146 (44.5%)	181 (55.5%)	1		1	
	Yes	29 (55.8%)	23 (44.2%)	1.56 (0.8, 2.82)	.137	1.25 (0.54, 2.87)	.604

Given this fact, female participants were 2.6 times (AOR = 2.56; 95% CI = 1.50–4.26; *P* <.001) more likely to experience depressive symptoms than male participants. It was also identified that participants who had a long duration of stay in the refugee camp (≥11 years) were 2.3 times more likely (AOR = 2.32; 95% CI = 1.39–3.86; *p* = .001) to have increased odds of depressive symptoms than those who had a short duration of stay. Similarly, refugees who have no job had increased odds of depressive symptoms by 2.7 times (AOR = 2.68; 95% CI = 1.30–5.50, *p* = .007) than those employed. Furthermore, participants with poor social support were three times (AOR = 3.12; 95% CI = 1.25–7.79; *p* = 0.015) more likely to experience depressive symptoms than those with good social support.

### Factors associated with anxiety symptoms

Factors like age, sex, educational status, marital status, family history of psychiatric illness, and social support fulfilled the minimum criteria (*p* < 0.25) for further multivariable logistic analysis. These variables were then entered into a multivariate logistic regression model (**Table 5**).

**Table 5:** Bivariate and multivariable analysis for all candidate factors for anxiety among Sudanese refugees at Bambasi Camp in northwest Ethiopia (*n* = 379).

Variable	Category	Anxiety		COR and 95% CI	*p*-value	AOR and 95% CI	*p*-value
		Yes	No				
Sex	Male	43 (25%)	129 (75%)	1		1	
	Female	107 (51.7%)	100 (48.3%)	3.21 (2.06, 4.98)	.000	3.60 (2.26, 5.74)	.000
Age	18–25	33 (32.4%)	69 (67.6%)	1		1	
	26–35	50 (39.4%)	77 (60.6%)	1.35 (0.78, 2.34)	.273	1.08 (0.60, 1.94)	.775
	36–45	40 (38.5%)	64 (61.5%)	1.30 (0.73, 2.31)	.360	1.05 (0.57, 1.94)	.863
	>45	27 (58.7%)	19 (41.3%)	2.97 (1.44, 6.09)	.003	2.48 (1.16, 5.30)	.019
Marital status	Married	59 (39.6%)	90 (60.4)	1		1	
	Single	49 (43%)	65 (57%)	1.15 (0.70, 1.89)	.580	1.38 (0.80,2.38)	.234
	Separated	22 (31.9%)	47 (68.1%)	0.71 (0.39, 1.30)	.274	.64 (0.33, 1.23)	.186
	Divorced	6 (26.1%)	17 (73.9%)	0.53 (0.20, 1.44)	.219	.64 (0.23,1.83)	.411
	Widowed	14 (58.3%)	10 (41.7%)	2.14 (0.89, 5.12)	.089	1.74 (0.68,4.46)	.246
Social support	Strong	11 (29.7%)	26 (70.3%)	1		1	
	Moderate	67 (40.6%)	98 (59.4%)	1.62 (0.75, 3.49)	.222	1.44 (0.64, 3.25)	.382
	Poor	72 (40.7%)	105 (59.3%)	1.62 (0.75, 3.48)	.217	1.01 (0.44, 2.31)	.987
Family mental illness	No	123 (37.6%)	204 (62.4%)	1		1	
	Yes	27 (51.9%)	25 (48.1%)	1.79 (0.99, 3.23)	.052	1.33 (0.67, 2.66)	.418

The finding from this study shows that female participants are 3.6 times (AOR = 3.60; 95% CI = 2.26–5.74; *p* <.001) more likely to experience anxiety symptoms compared to male participants. The study also reveals that those participants in the age category above 45 were 2.5 times (AOR = 2.48; 95% CI = 1.16–5.30, *p* = .019) more likely to show anxiety symptoms compared to the younger age (18– 25) group.

## Discussion

The main aim of this study is to assess the prevalence and associated factors of depressive and anxiety symptoms among Sudanese refugees at Bambasi Camp. Our analysis found that the prevalence of depressive and anxiety symptoms in the Bambasi Camp was 46.2% (95% CI: 41–51%) and 39.6% (95% CI: 34.2–41.2), respectively.

The magnitude of depressive symptoms in our finding is in line with previous studies done in Uganda at 46.2% (32), southern Juba at 49.9% (12), Syrian refugees in Greece at 44% (33), Karenina refugees living in the Thai–Burma border at 41.8% (34), and USA at 47.7% (35). However, it was higher in comparison to the previous studies done on Somali and Eritrean refugees in Ethiopia at 38.3% and 37.8%, respectively (14, 36), northern Uganda at 15.2% (37), Somali refugees in Kenya at 40.8% (29), Istanbul at 34.7% (38), Germany at 39.8% (39), and Burmese refugees in Australia at 36% (40). The possible explanation could be the ongoing feeling of insecurity in their home country (Sudan), and the sensitivity of the tool differences may be the reason for the discrepancy—for instance, in a study among Eritrean and Somali refugees’ camps in Ethiopia that used PHQ 9, we used HSCL-25. In addition, refugees in low-income countries have increased odds of depression due to multiple reasons (41). Conversely, the prevalence of depressive symptoms in the current study is lower than in a study conducted among African refugees in South Africa at 54.6% (16), Syrian refugees residing in Iraq at 59.4% (42), Cambodian refugees in the USA at 80% (43), Canada at 51.7% (44), and Bangladesh at 51.6% (45). The possible explanation might be due to the use of different tools in Afghanistan and USA; both used DASS-21 and Composite International Diagnostics Interview, respectively. Furthermore, sampling procedures, study design, cultural differences among refugees, and the use of a large sample size may amplify the prevalence.

The prevalence of anxiety symptoms in our study revealed 39.6% (95% CI: 34.2–41.2), which is in line with research done in Istanbul at 36.1% (38), USA at 40.3% (35), and Karenni refugees in Thai–Burmese at 41% (34). However, the current prevalence study was higher than the study conducted in Germany from Arabic-speaking refugees at 26.8% (46), Syrian refugees in Germany at 13.5% (47), and Australia at 20% (40). The possible explanation for this may be separation anxiety, the type of stress experienced during the post-migration period, and also the added load of resettlement procedures in a new country (48). In addition, the use of a 3-year follow-up study design in Germany, the use of the DASS 21 tool, and a smaller sample size (*n* = 148) in Australia may be the possible explanations. Conversely, the prevalence of anxiety in the current study is lower than in a study done in Bangladesh at 70% (45), Afghanistan at 72.2% (49), South Africa at 49.4% (16), and Uganda at 73% (17). This variation across these settings could be due to various reasons, such as the use of different tools, cultural differences in illness narratives, sampling techniques, and types of trauma experienced (50).

In our study, being female was associated with depressive symptoms. The finding shows that female participants have 2.6 times more chances of experiencing depressive symptoms than male participants. This finding is consistent with other studies done in Tigray (14), Somalia refugees in Ethiopia (36), Somalia refugees in Kenya (29), Greece (33), Ukrainian refugees in Greece (51), Syrian refugees residing in the Kurdistan region of Iraq (42), and Yazidis refugees in Turkey (52). However, in contrast to this, research done in Uganda shows that female participants are equally affected as men (32), and among Rohingya refugees in Bangladesh, Ethiopian refugees in Toronto, and the conflict-affected population in Somalia, women were less likely to get depression than men (41, 45, 53), respectively. The possible reason for this discrepancy might be that, in the war zone, women faced gender-specific risks as potential victims of rape, sexual abuse, targeted killing, widowhood of deceased soldiers, and pregnancy-related complications due to poor antenatal and postnatal healthcare (54). Additionally, despite the complexity of the sex difference in depression, recent evidence suggests that biological factors, particularly decreases in estrogen during menses, lactation, and menopause, may contribute to the increased prevalence of depression in women (3). On the contrary, testosterone conversion in male patients’ brain via aromatase, presence of androgen receptors in hippocampal neurons, non-recycling nature of testosterone in male patients, and presence of sexually dimorphic brain nuclei in male patients convey special protection for depression among male (55).

Our finding reveals that participants who have no job in the camp are significantly associated with depressive symptoms. Several factors contribute to this association. This study is supported by a study conducted on Eritrean refugees in Tigray (14), Afghanistan post-war (49), Somalia (53), and Rohingya refugees in Bangladesh (45). The possible explanation for these might be that unemployment can lead to feelings of hopelessness, loss of purpose, and reduced self-esteem and can also hinder the refugees’ ability to meet their basic needs and engage in meaningful activities. This leads to social isolation, dependency on aid, and limited opportunity for personal growth and development, which can contribute to feelings of worthlessness and depression (10).

In addition, staying 11 and more years in the refugee camp was a significant predictor of depressive symptoms. This is supported by a study conducted on Eritrean refugees in Tigray (14), Moria refugee camp in Greece (56), and in Canada (57). The possible reason for this might be the limited opportunities for education, employment, and mental health services in the camp, and integration in camp settings can exacerbate feelings of frustration, stagnation, and dependency (56). Over time, the initial support from fellow refugees or external aid organizations may decline, leaving long-term refugees more vulnerable to feelings of loneliness, uncertainty for the future, and disconnection from their homeland and cultural roots (58).

In this study, participants who had poor social support were more likely to increase the odds of getting depressive symptoms than those who had good social support. Our study is in line with the studies done in Tigray, Bangladish, Turkey, and Sudan. The possible reason might be poor social support among refugees, which can contribute to depression through mechanisms such as social isolation, limited access to resources, and recovery from trauma and stress (41).

Being female was another significant predictor for anxiety symptoms. This finding is consistent with studies done in Istanbul (38), Norway (59), and Bangladesh (45). The possible explanation may be that women in refugee camps often have a higher risk of gender-based violence, including sexual assault, domestic violence, and exploitation (60). Moreover, female patients may experience limited access to healthcare services, including mental health support, due to overcrowding and a lack of privacy and confidentiality in seeking healthcare services, which increases the level of stress and anxiety among women (61).

Additionally, our analysis showed that age above 45 was associated with anxiety symptoms, in line with a study conducted in Thailand (62), and Australia (63). The reason might be that refugees over 45 experience unique stressors related to their age, for example, declining physical health, reduced social support networks, acculturation, loss of social roles, and integration into the host society as well as an increased feeling of anxiety and worry about their health (64).

This study is not without limitations. The following are potential limitations: Due to the nature of sensitivity and recall bias, there may be underreporting of traumatic event items. HSCL-25 is a screening tool rather than a diagnostic tool, so the endpoint of depressive and anxiety symptoms might not be certain. Additionally, using self-report data, cross-sectional study design, limited socioeconomic data, and language and cultural barriers.

## Conclusion

This study revealed that nearly half and more than one in three of the participants had depressive and anxiety symptoms, respectively. Being female heightened the risk of experiencing depressive and anxiety symptoms. This study recommends routine screening for depression and anxiety in the refugee camp setting. Additionally, community support networks including the host community can be fostered by organizing support groups and peer counseling programs that promote social cohesion and resilience, moreover facilitating conditions for the inclusion with the Ethiopian host society, and involving them in socio-economic participation or sustaining a meaningful resolution to the conflict in Sudan would facilitate the return of refugees back to their homes, support the healing process, and re-build their lives as recommended.

## Data Availability

The original contributions presented in the study are included in the article/supplementary material. Further inquiries can be directed to the corresponding author.
